# „Time in range“ (TIR) vs. Glykohämoglobin Typ A_1c_ (HbA_1c_): was zählt für unsere Patienten? Metriken der kontinuierlichen Glukosespiegelmessung in der Praxis

**DOI:** 10.1007/s11428-022-00963-9

**Published:** 2022-10-11

**Authors:** Clemens Harer, Julia K. Mader

**Affiliations:** Universitätsklinik für Innere Medizin, Abteilung für Endokrinologie und Diabetologie, Medizinische Universität Graz, Auenbruggerplatz 15, 8036 Graz, Österreich

**Keywords:** Diabetes mellitus, Glykämische Kontrolle, Lebensqualität, Telemedizin, E‑Health, Diabetes mellitus, Glycemic control, Quality of life, Telemedicine, eHealth

## Abstract

„Continuous glucose monitoring systems“ (CGM-Systeme) und CGM-basierte Metrik gewannen in den letzten 10 Jahren massiv an Bedeutung. Dennoch ist der HbA_1c_ nach wie vor der meistverwendete und international anerkannte Marker zur Beurteilung der glykämischen Kontrolle. Ebenso stellt er in klinischen Studien immer noch den wichtigsten Surrogatparameter zur Beurteilung klinischer Outcomes dar. Die Verwendung der Zeit im Zielbereich („time in range“ [TIR]) hat im Vergleich zum HbA_1c_ den Vorteil, dass Hypoglykämien und Glukosevariabilität besser dargestellt werden. Durch Nutzung der TIR kann man auch individuelle Zielbereiche definieren, beispielsweise bei Schwangeren oder multimorbiden Personen. Auch gibt es erste Hinweise, dass klinische Studienergebnisse anhand von TIR und anderen CGM-basierten Metriken bewertet werden können, auch wenn hierzu noch Langzeit- und Endpunktstudien fehlen. Einen wesentlichen Vorteil zeigt die TIR bei der Prädiktion diabetesassoziierter Komplikationen. So kann, basierend auf Änderungen beim erreichten Zielbereich, nicht nur das Auftreten neuropathischer, mikro- oder makrovaskulärer Komplikationen vorhergesagt werden, sondern auch das relative Risiko deren Manifestation. Die Nutzung von CGM im Allgemeinen und das Erreichen der TIR-Ziele spielen auch für Menschen mit Diabetes mellitus und deren Einschätzung ihrer Lebensqualität eine immer größere Rolle.

## Abstract (en)

The use of continuous glucose monitoring (CGM) systems and CGM-based metrics has increased massively in recent years in clinical care. However, HbA_1c_ remains the most widely used parameter to assess glycemic control. Even despite its limitations, HbA_1c_ is still the most commonly used parameter for glycemic control and outcome in clinical trials. Time in range (TIR) as a measure of CGM has many advantages over HbA_1c_, since it provides deeper insights into glucose variability and detection of hypoglycemia. In addition, individual TIRs can be applied, for example, in pregnant women or patients with comorbidities. TIR is a powerful tool to evaluate clinical outcomes in diabetes trials; however, standardized methods are still lacking. Changes in TIR furthermore predict the risk of diabetes complications based on the achievement of TIR-based targets. Use of CGM has improved quality of life in people living with diabetes. In recent years people living with diabetes are more familiar with the importance of TIR-based metrics as parameters of glucose control.

In der Praxis kann es sich für das betreuende Gesundheitspersonal als schwierig erweisen, HbA_1c_ und TIR gegeneinander abzuwägen, denn die CGM-basierten (CGM: „continuous glucose monitoring“) Metriken sind noch relativ neu, und nicht jeder kennt deren Bedeutung. Die wichtigsten CGM-basierten Metriken und die Vorteile der zielwertbasierten Glukosevariabilitätskontrolle werden vorgestellt und Letztere mit gängigen Parametern der glykämischen Kontrolle verglichen. Zudem wird die künftig größere Rolle der TIR-Metriken erläutert und dargelegt, warum diese auch im klinischen Alltag Einzug halten sollten.

## Geschichte der Langzeitglukosebestimmung

### Glykohämoglobin (HbA_1c_)

- 1966 entdeckten Holmquist und Schroeder 5 Subtypen von HbA, darunter auch HbA_1c_.
- 1968 entdeckte Rahbar, dass HbA_1c_ bei Menschen mit Diabetes mellitus erhöht ist.
- 1975 wurde HbA_1c_ erstmals von Koenig und Cerami als Indikator für die Glukosekontrolle postuliert.
- 1993 wurde HbA_1c_ aufgrund der Ergebnisse des „diabetes control and complications trial“ (DCCT) als Indikator für Menschen mit Typ-1-Diabetes zugelassen.
- 1998 wurde HbA_1c_ aufgrund der Resultate der „UK prospective diabetes study“ (UKPDS) auch für Menschen mit Typ-2-Diabetes zugelassen.
- 2010 wurde die Zulassung durch eine Empfehlung der American Diabetes Association (ADA) unterstrichen [1].

Der HbA_1c_ ist weiterhin der meistetablierte Marker in den internationalen Leitlinien zur Beurteilung der glykämischen Kontrolle, Abschätzung des Risikos für Begleiterkrankungen und Therapieempfehlungen [2]. Er ist auch weiterhin der Hauptsurrogatparameter für die Beurteilung des Outcomes in klinischen Studien [3].

### Kontinuierliche Glukosespiegelbestimmung (CGM)

- 1999 wurde CGM erstmals zur Messung subkutaner Glukosespiegel eingesetzt [4].
- 2012 veröffentlichte das International Diabetes Center (IDC) Helmsley die erste Konsensusrichtlinie zur Standardisierung von CGM-Metriken [5].
- 2013 erfolgte der internationale Konsens zur Verwendung des AGP-Berichts (AGP: „ambulatory glucose profile“, [6]).
- 2017 wurden erstmals die internationalen Zielwerte für TIR festgelegt.
- 2019 gab die ADA, nach weiteren Anpassungen der Metriken, generelle Empfehlungen für die Verwendung und Ziele der TIR ab [8, 9].

In den letzten Jahren hat die Anzahl der verwendeten CGM-Systeme rapide zugenommen (von 7 % bis 2012 auf 30 % bis 2018), was insbesondere auf die verringerten Kosten sowie die einfachere Handhabung zurückzuführen ist [10, 11]. Obwohl CGM-Systeme mittlerweile weit verbreitet sind, fehlt immer noch die Standardisierung von Auswertungsmethoden zu deren Nutzung in klinischen Studien [3].

## Limitierungen von Glykohämoglobin als Parameter der Glukosespiegelkontrolle

**Figure 1:**
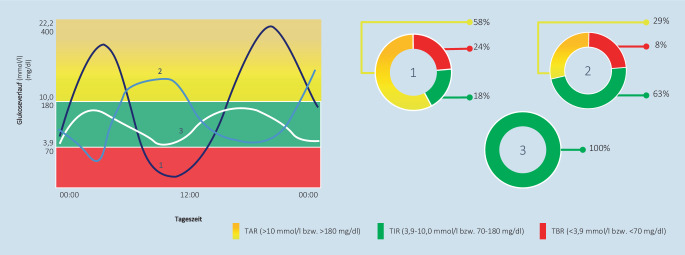

Der HbA_1c_ eignet sich gut zur Bewertung des Auftretens von Hyperglykämien über einen Zeitraum von mehreren Wochen, liefert jedoch wenig Informationen hinsichtlich des Glukosespiegelverlaufs über den Tag, dessen Muster oder die Glukosevariabilität [12]. Somit können Personen mit dem gleichem HbA_1c_ beispielsweise ganz unterschiedliche Glukoseverläufe über 24 h aufweisen ([13]; Abb. 1). Dagegen lässt sich mittels CGM-Systemen die glykämische Variabilität bei Personen mit Diabetes mellitus und dem gleichen HbA_1c_ ermitteln. Sie ermöglichen somit eine Quantifizierung der Unterschiede unter Nutzung von TAR („time above range“), TIR und TBR („time below range“), um bessere klinische Entscheidungen treffen zu können.

Außerdem gibt es relevante Faktoren, die unabhängig von der Glykämie den HbA_1c_ beeinflussen, wie Hämoglobinopathien, chronische Niereninsuffizienz oder hämolytische Anämie [14]. Ethnische Unterschiede und Heterogenität in der Lebensdauer von Erythrozyten können ebenso wesentliche Auswirkungen auf den HbA_1c_ haben [15, 16].

## Kontinuierliche Glukosespiegelbestimmung und Zeit im Zielbereich – Definitionen

### Arten der kontinuierlichen Glukosespiegelbestimmung

Die CGM-Systeme werden in 3 Kategorien eingeteilt:

- „intermittent scanning CGM“ (isCGM): manueller Sensorscan, auch Flashsysteme genannt
- retrospektive CGM (rCGM): erst retrospektiv auswertbar
- „real time CGM“ (rtCGM): Echtzeitmessung

Sie haben unterschiedliche Vor- und Nachteile, wobei rCGM jedoch nur selten verwendet werden. Die Nutzung von isCGM-Systemen geht mit einer Verkürzung der Hypoglykämiedauer bei Menschen mit Typ-1- und Typ-2-Diabetes einher [17, 18], und sowohl mit Insulinpumpen- als auch mit Basis-Bolus-Therapie mittels Pen versorgte Personen profitieren gleichermaßen von der rtCGM-Verwendung. Insgesamt kam es dabei zu einer Reduktion sowohl der Häufigkeit als auch der Schwere von Hypoglykämien bei gleichzeitig verbesserter glykämischer Kontrolle [7].

### Metriken der Zeit im Zielbereich

Neben anderen Parametern, wie durchschnittliche Glukosekonzentration, AGP-Profil, geschätztem HbA_1c_ (eA_1c_: „estimated“ A_1c_) bzw. Glukosemanagementindikator (GMI), Sensornutzungszeit und Anzahl an Scans, liegt der Fokus bei der Beurteilung der glykämischen Kontrolle insbesondere bei der Bewertung der Zeiten im Zielbereich. Diese setzen sich aus folgenden Subgruppen zusammen (Angaben: Sensorglukose in mmol/l bzw. mg/dl, jeweiliger Zielwert in %):

- „Time above range“ (TAR): < 25 %
  - Stufe-1-Hyperglykämie: > 10,0–13,9 mmol/l bzw. > 180–250 mg/dl
  - Stufe-2-Hyperglykämie: > 13,9 mmol/l bzw. > 250 mg/dl, < 5 %
- „Time in range” (TIR): 3,9–10,0 mmol/l bzw. 70–180 mg/dl, > 70 %
- „Time below range” (TBR): < 4 %
  - Stufe-1-Hypoglykämie: < 3,9–3,0 mmol/l bzw. < 70–54 mg/dl
  - Stufe-2-Hypoglykämie: < 3,0 mmol/l bzw. < 54 mg/dl, < 1 %

**Figure 2:**
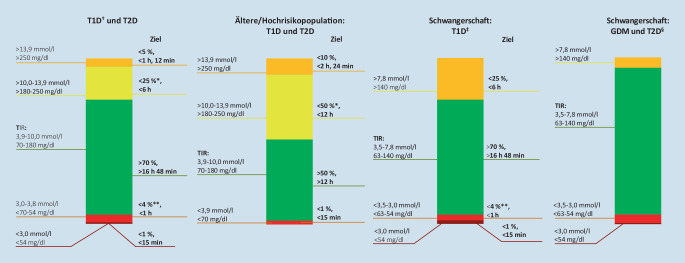

Die Subklassifizierung der Zeit bezüglich Hypo- und Hyperglykämien wurde eingeführt, um damit Risiken für schwere Hypoglykämien bzw. diabetische Spätkomplikationen und Ketoazidose besser bewerten zu können. Die Darstellung der Zielwerterreichung kann einerseits in %, andererseits in durchschnittlichen Stunden im Zielbereich angegeben werden ([8, 19]; Abb. 2).

Während der Schwangerschaft gelten, neben spezifischen Vorgaben zur Nüchternglukose- (< 5,3 mmol/l bzw. < 95 mg/dl), 1‑h-postprandialen Glukose- (< 7,8 mmol/l bzw. < 140 mg/dl) und 2‑h-postprandialen Glukosekonzentration (< 6,7 mmol/l bzw. < 120 mg/dl), eigene Metriken der TIR:

- TAR: > 7,8 mmol/l bzw. > 140 mg/dl, < 25 %
- TIR: 3,5–7,8 mmol/l bzw. 63–140 mg/dl, > 70 %
- TBR: < 4 %
  - Stufe-1-Hypoglykämie: < 3,5–3,0 mmol/l bzw. < 63–54 mg/dl
  - Stufe-2-Hypoglykämie: < 3,0 mmol/l bzw. < 54 mg/dl, < 1 %

Hierbei sei angemerkt, dass bisher wenige aussagekräftige Daten zur Definition der TIR bei Schwangeren mit Typ-2-Diabetes oder bei Schwangerschaftsdiabetes vorliegen ([8, 20, 21]; Abb. 2).

## Bedeutung der Zeit im Zielbereich

### Rolle als Prädiktor diabetesbezogener Komplikationen

Beck et al. [12] stellten fest, dass eine vorbekannte Retinopathie um 64 % und eine Mikroalbuminurie um 40 % für jede 10 %-Stufe, die sich die TIR_70–180_ _mg/dl_ reduziert, voranschreiten. Personen mit niedrigerer TIR wiesen außerdem eine wesentlich höhere Intima-Media-Dicke der Karotiden auf [22]. Auch bezüglich der Prävalenz der diabetischen Polyneuropathie wurde festgestellt, dass diese Komplikation bei einer TIR_70–180_ _mg/dl_ < 70 % signifikant häufiger auftrat [23]. Laut Ergebnissen der DEVOTE-Studie können die „hazard ratios“ für MACE („major adverse cardiovascular events“) bei Personen mit Typ-2-Diabetes und hohem Risiko für kardiovaskuläre Erkrankungen nicht nur bei einer TIR_70–180_ _mg/dl_ > 70 % um 30 % verringert werden, zudem bewirkt eine TIR_70–180_ _mg/dl_ von 50–70 % lediglich eine 9 %ige Verringerung im Vergleich zu einer TIR_70–180_ _mg/dl_ ≤ 50 %. Außerdem ist die TIR_70–180_ _mg/dl_ mit der Zeit bis zur ersten schweren Hypoglykämie sowie dem Auftreten von mikrovaskulären Ereignissen assoziiert [24]. Die wichtigsten Auswirkungen der TIR_70–180_ _mg/dl_ auf die 10-Jahres-Wahrscheinlichkeit für diabetesassoziierte Komplikationen finden sich in Tab. 1, basierend auf dem „IQVIA core diabetes model 2019“ [25].

**Table 1:** 

Komplikation	TIR 80 %	TIR 70 %	TIR 58 %
	T1D | T2D	T1D | T2D	T1D | T2D
Herzversagen	0,45–0,55 | 7,43–7,52	0,55–0,61 | 7,51–7,58	0,68 | 7,55
Herzinfarkt	2,25–2,70 | 11,37–11,97	2,65–2,97 | 11,99–12,39	3,29 | 12,76
Schlaganfall	0,38–0,47 | 6,74–7,03	0,45–0,51 | 6,97–7,14	0,57 | 7,37
Mikroalbuminurie	1,56–3,23 | 10,26–12,63	3,07–3,23 | 12,54–14,05	6,90 | 15,85
pAVK	1,59–1,97 | 7,29–8,15	1,95–2,16 | 8,11–8,54	2,46 | 9,05
Retinopathie	1,46–3,10 | 0,68–0,88	2,92–4,41 | 0,88–1,02	6,67 | 1,21
Neuropathie	5,24–8,65 | 20,04–24,11	8,32–10,93 | 23,76–26,13	14,38 | 28,84
Ulkus	1,38–1,92 | 0,92–1,03	1,83–2,43 | 1,01–1,06	3,00 | 1,12

*pAVK* periphere arterielle Verschlusskrankheit, *T1D* Typ-1-Diabetes, *T2D* Typ-2-Diabetes, *TIR* „time in range“

Aber auch in der Schwangerschaft erhöht eine Reduktion der TIR um 5–7 % das Risiko für Makrosomie, Schulterdystokie, Intensivstationsaufhalte und neonatale Hypoglykämie signifikant [26].

### Lebensqualität bei Überwachung der Therapieziele anhand der Zeit im Zielbereich

Bei der Beurteilung der Lebensqualität (QoL) spielen verschiedene individuelle Faktoren, aber auch Unterschiede zwischen den Diabetestypen eine Rolle. In einer Studie stellte sich heraus, dass die Mahlzeitenwahl für sämtliche Diabetestypen von größter Bedeutung war (63–67 % der Befragten). Jedoch berichteten beispielsweise Menschen mit Typ-1-Diabetes, dass die Erreichung der TIR für sie den zweitgrößten Einfluss auf das tägliche Leben hat (57 % der Befragten), während Hypoglykämien und HbA_1c_ von jeweils nur 30 % dieser Gruppe als wichtige Faktoren beschrieben wurden. Bei Menschen mit Typ-2-Diabetes wurde der HbA_1c_ jedoch mit der TIR an zweiter Stelle gleichgestellt (41–45 %). Menschen mit Diabetes mellitus, aber ohne Insulintherapie, bewerteten beide Parameter sogar tendenziell als etwas weniger bedeutsam [27].

### Glukosevariabilität und ihre Bedeutung für klinische Studien

Laut Bestimmungen in klinischen Studien kann die TIR bei Typ-1- und Typ-2-Diabetes in eindeutiger Relation zu etablierten Parametern der Glukosekontrolle gesehen werden. Eine TIR von 70 % entspricht laut aktuellen Untersuchungen einem HbA_1c_ von 7 % bzw. 53 mmol/mol [29, 30, 33]. Jedoch besteht zwischen den Parametern eine inverse Korrelation [28, 29]. Aber auch bezüglich glykiertem Albumin, welches im Vergleich zum HbA_1c_ sensitiver auf die postprandiale Glukosekonzentration reagiert, wurde eine ähnliche Korrelation nachgewiesen [30]. Außerdem stellte sich heraus, dass sich die TIR in klinischen Studien genauso gut eignet, das Risiko von Retinopathie und Mikroalbuminurie zu stratifizieren, wie der HbA_1c_. Anders als beim HbA_1c_ wurde zudem nachgewiesen, dass sich die durchschnittliche Glukosekonzentration anhand der TIR präziser vorhersagen lässt, da sie, wie eingangs erwähnt, die Glukosevariabilität besser abbildet [12].

Nachdem auch in Zukunft zumindest ein gewisser Anteil an Personen mit Diabetes mellitus CGM nicht oder nur eingeschränkt wird nutzen können, ist es nötig, künftig Studienmodelle zu schaffen, welche Patienten sowohl mit als auch ohne CGM-Nutzung berücksichtigen.

### Metriken der Zeit im Zielbereich im Alltag und Ausblick

Obwohl die Verwendung von CGM-Systemen längst in den Alltag von Menschen mit Diabetes mellitus Einzug gehalten hat und zur aktuellen Therapieentscheidung genutzt wird, bestehen immer noch Hürden bei der Verwendung und Interpretation von CGM-basierten Metriken durch Gesundheitspersonal, aber auch Betroffene. Es ist daher dringend notwendig, klare und einfache Anleitungen sowie anwenderfreundliche Systeme zu entwickeln, damit diese Verfahren optimal genutzt werden können. Ein Vorschlag wäre beispielsweise, einen definitiven Workflow vorzugeben, in dem die 10 wichtigen Punkte der CGM-Metriken der Reihe nach abgearbeitet werden. In weiterer Folge bedarf es auch der Schaffung eines neuen Bewusstseins der Menschen mit Diabetes mellitus für die regelmäßige Datenauswertung ihrer CGM-Systeme sowie auch der Aufklärung und Unterstützung durch das Gesundheitspersonal. Regelmäßige Datenanalyse durch die Betroffenen selbst ermöglicht rasche, eigenständige Therapieanpassungen, ohne auf den 3‑monatlichen HbA_1c_-Wert zu warten [31].

Regelmäßige CGM-Daten-Analyse durch die Betroffenen erlaubt rasche, eigenständige Therapieanpassungen

Laut Untersuchungen aus dem „IQVIA diabetes core model“ werden bei Menschen mit Typ-1-Diabetes die Kosten für das Gesundheitssystem bei einer TIR_70–180_ _mg/dl_ zwischen 70 und 80 % um 50 % reduziert. Auch wenn der relative Unterschied bei Menschen mit Typ-2-Diabetes nicht so groß ist [31], wäre die Einsparung in absoluten Zahlen aufgrund des größeren Kollektivs bei weiter verbreiteter Verwendung vermutlich noch größer.

In den Jahren 2020–2022 gewann die telemedizinische Betreuung von Menschen mit chronischen Erkrankungen, darunter auch Diabetes mellitus, aufgrund der COVID-19-Pandemie (COVID-19: „coronavirus disease 2019“) zunehmend an Bedeutung und hielt auch Einzug in die Diabetesversorgung. Die server- oder cloudbasierte elektronische Übertragung diabetesbezogener Daten ermöglicht bis zum heutigen Tag, dass Menschen mit Diabetes mellitus und Gesundheitspersonal aktuelle CGM-Daten – und nach Möglichkeit auch die der Insulindosierung – in gewohnter Qualität, kontaktlos und ohne umständliche (teils analoge) Übermittlung von Einzelergebnissen, einsehen und besprechen können. Bisher etablierte telemedizinische Versorgungsmodelle werden jedoch weiterentwickelt werden müssen, da im bisherigen Setting die Gefahr der verzögerten Erstdiagnosestellung sowie eine höhere Wahrscheinlichkeit für das Auftreten einer diabetischen Ketoazidose bestehen [32].

Grundlegende Erkenntnisse einer Pilotstudie zeigten auch das Anwendungspotenzial von CGM-Metriken im stationären Setting, wo diese Systeme, insbesondere aufgrund fehlender Zulassung, bisher kaum etabliert sind. Es wurde nachgewiesen, dass Effekte auf TIR-Metriken im stationären Bereich in erster Linie vom Diabetestyp und Aufnahmegrund (akut oder elektiv zu Schulungszwecken) abhängen. So hatten Menschen mit Typ-2-Diabetes und jene mit akuter Aufnahmeindikation bei der Entlassung eine niedrigere TAR zugunsten einer höheren TIR. Zu Schulungszwecken Aufgenommene verbesserten zwar ihre TBR, jedoch zu Lasten einer höheren TAR, ähnlich Menschen mit Typ-1-Diabetes, bei denen tendenziell eine Zunahme in beiden Metriken (TAR und TBR) festzustellen war. Gruppenabhängige Einstellung bei der Aufnahme, pathognomonische Glukosevariabilitätsunterschiede zwischen den Diabetestypen, vorbestehende Insulintherapie oder CGM-Nutzung sind hierbei vermutete Faktoren, welche den Verlauf beeinflusst haben könnten. Subjektive Zeitersparnis für das Gesundheitspersonal und tendenziell größere Patientenzufriedenheit sind Aspekte, welche in Zukunft unbedingt beachtet werden sollten [33].

## Fazit für die Praxis

- Im Gegensatz zum HbA_1c_ (Glykohämoglobin) geben TIR („time in range“), TAR („time above range“) und TBR („time below range“) gemeinsam mit dem AGP („ambulatory glucose profile“) Rückschlüsse über Muster der glykämischen Kontrolle, Auftreten und Schwere von Hypoglykämien sowie glykämische Variabilität.
- Die TIR korreliert negativ mit dem Auftreten diabetischer Langzeitkomplikationen und Komplikationen in der Schwangerschaft.
- Das Erreichen einer TIR_70–180_ _mg/dl_ > 70 % und eine TBR_<_ _70_ _mg/dl_ < 4 % bzw. TBR_<_ _54_ _mg/dl_ < 1 % (Stufe 2) werden zur Vermeidung diabetischer Komplikationen empfohlen.
- Die TIR könnte dem HbA_1c_ als Surrogatparameter in klinischen Studien überlegen sein, allerdings fehlen dazu noch Langzeit- und Outcomestudien.
- Auf kontinuierlicher Glukosespiegelmessung (CGM) basierte Metriken spielen in der Diabetesversorgung inklusive Nutzung in der Telemedizin eine wichtige Rolle.
- In Zukunft könnte auch die Nutzung CGM-basierter Metriken im stationären Setting von Bedeutung sein.
